# A novel multiplex PCR method for the detection of virulence-associated genes of *Escherichia coli* O157:H7 in food

**DOI:** 10.1007/s13205-015-0319-0

**Published:** 2015-12-31

**Authors:** Vo Van Giau, Thuy Trang Nguyen, Thi Kim Oanh Nguyen, Thi Thuy Hang Le, Tien Dung Nguyen

**Affiliations:** 1 Deparment of Faculty of Food Technology, Ho Chi Minh City University of Food Industry (HUFI), 140 Le Trong Tan, Tan Phu District, Ho Chi Minh City, Vietnam; 2 Gachon Medical Research Institute, Gachon University, Seongnam, South Korea; 3 Faculty of Pharmacy, University of Medicine and Pharmacy, Ho Chi Minh City, Vietnam; 4 Laboratory of Microbiology, National Agro- Forestry- Fisheries Quality Assurance Department Branch 4 (NAFIQAD 4), 30 Ham Nghi, District 1, Ho Chi Minh City, Vietnam

**Keywords:** Multiplex PCR, Validation, *E. coli* O157:H7, stx1 and stx2

## Abstract

Shiga toxin-producing *Escherichia coli* O157:H7 (*E. coli* O157:H7) strains are foodborne infectious agents that cause a number of life-threatening diseases, including hemorrhagic colitis (HC) and hemolytic uremic syndrome (HUS). Shiga toxin 1 (stx1), shiga toxin 2 (stx2), or a combination of both are responsible for most clinical symptoms of these diseases. Hence, various diagnostic methods have been developed so far to detect shiga toxins such as cell culture, ELISA, Rapid Latex Agglutination (RPLA) and hybridization, but due to high costs and labor time in addition to low sensitivity, they have not received much attention. The aim of this study was to develop a complete, rapid and reliable multiplex PCR (mPCR) method by using two pairs of specific primers to detect either the *stx1* or the *stx2* gene confirms the presence of *E.coli* O157:H7. The study results show that *stx*1F/*stx*1R primers are specific for *stx1* and primers *stx*2F/*stx*2R are specific for *stx2* genes in *E. coli* O157:H7. The mPCR method with two pairs of primers for amplifying the *stx*1, *stx*2 target genes to detect *E. coli* O157:H7 in food has been set up successfully. Complete method performed well in both types of food matrices with a detection limit of 3 CFU/25 g or mL of food samples. Tests on 180 food samples have shown a specificity value of 93.75 % (95 % confidence interval [CI], 82.83–100), a sensitivity of 100 % (95 % CI, 83.79–99.85 %), and an accuracy of 96.66 % (CI 95 %, 83.41–99.91 %). Interestingly, results indicate that the mPCR performed as well as the traditional culture methods and can reduce the diagnosis time to 2 days. Finally, complete mPCR method was applied to natural samples covering a wide variety of food types proving that the mPCR method was a rapid and reliable screening method for detection of *E. coli* O157:H7 in food and environmental samples.

## Introduction

The best-known and also most notorious *E. coli* bacteria that produce Shiga toxin (STEC) is *E. coli* O157:H7 that caused foodborne diseases, usually hemorrhagic colitis (HC) and hemolytic uremic syndrome (HUS) (Karmali 1989, 2004). According to recent reports by the Center for Disease Control and Prevention (CDC 2014) there were 11 multistate outbreaks of STEC in the United States of America during 2011–2014 with six of them attributed to *E. coli* O157:H7. Furthermore, *E. coli* O157:H7 has been recognized as a major food safety concern due to its low infectious dose. It is highly virulent, an inoculation of fewer than 10–100 CFU of *E. coli* O157:H7 is sufficient to cause infection (Coffey et al. 2011). *E. coli* O157:H7 pathogens were usually linked with a wide variety of foods such as milk, meats, meat products, dairy products and fresh products, contaminated from the field with wildlife droppings (Bell 2002; Fedio et al. 2011; Mora et al. 2007; Pennington 2010). It is also reported that *E. coli* O157:H7 be cross-contaminated in the preparation of meat products from intestines (Wachtel et al. 2003).

Shiga toxin (Stx) is one of the major virulence factors involved in *E. coli* O157:H7 pathogenesis (Melton-Celsa et al. 2012); based on immunoreactivity, toxins are classified as either Stx1 or Stx2 (Strockbine et al. 1986), which damage intestinal epithelial cells and kidneys, causing HC and HUS, respectively (Johannes and Römer 2010). The stx gene in *E. coli* O157:H7 is associated with a prophage (Huang et al. 1987), and different subtypes of shiga toxin are identified as stx1, stx1c, stxfc, stx2, stx2e, stx2d and stx2g (Gobius et al. 2003). *Stx2* producing strains appear to be more commonly responsible for serious complications such as HUS than those only *Stx1* producing (Kleanthous et al. 1990; Read et al. 1992). Infections by STEC is a major health concern even developed countries as well as developing countries all over the world. Since shiga toxins cause many diseases, especially in children and immunocompromised elderly people, a rapid and sensitive diagnostic method with prognostic information would be rather useful. Isolation of *E.coli* O157:H7 from foods and fecal samples usually took few days with conventional diagnostic methods [ISO 16654 (2001)]. Conventional methods are laborious as they require the preparation of culture media, inoculation of plates and colony counting (Mandal et al. 2011). Furthermore, conventional methods may be limited by their low sensitivity (Lee et al. 2014). False-negative results may occur due to viable, but non-culturable pathogens. The failure to detect foodborne pathogens would increase the transmission risk of pathogens. So far, a variety of methodologies were developed to detect the presence of *E. coli* O157:H7; cell culture, serological, and Rapid Latex Agglutination (RPLA) have been utilized to detect shiga toxins or their respective genes. However, all these methods have their own shortcomings as they are time-consuming, quite costly and have limitations in handling many samples simultaneously. For cultivating samples in selective mediums, followed by latex agglutination or enzymelinked immunosorbent assay (ELISA) for confirming their subtype (*E. coli* Pro O157). Unfortunately, the detection limit from indirect assay was at 10^5^ cells/mL using antibodies, which was not enough to detect *E. coli* O157:H7 directly from food (Chart 1999). Rapid detection methods are important, particularly in food industry, as they are able to detect the presence of pathogens in raw and processed foods immediately. Rapid methods are also sensitive enough to detect pathogens that present in low numbers in the food. At present, molecular methods such as PCR are showing much promising results. In this situation, to overcome shortcomings of the aforementioned methods, mPCR is an appropriate alternative approach for detecting *stx* genes, improving both the sensitivity and specificity of the pathogen through amplification of its unique DNA. The mPCR is more convenient for rapid and reliable detection and quantification of pathogen-specific gene sequences.

The aim of the present work was the development of a complete multiplex PCR method for the detection of *E.coli* O157:H7 by using two pairs of specific primers to detect either the *stx1* or the *stx2* gene. Furthermore, the mPCR method is an appropriate alternative approach for detecting *stx* genes, able of achieving a low limit of detection, even in the presence of high numbers of competitors.

## Materials and methods

### Bacterial strains

*E. coli* O157:H7 strains used in this study are listed in Table 1. Three strains, one produces both Stx1 and Stx2, and one produces Stx1 only, one produces Stx2 only, were obtained from American Type Culture Collection (ATCC). In addition, these bacterial other pathogenic strains were approved by the National Agro-Forestry and Fisheries Quality Assurance Department Branch 4 (NAFIQAD 4); and eight *E. coli* strains (E1–E8) were isolated from retail beef, fork, vegetables and their geographical location was originated from Ho Chi Minh city area. The strains were verified by biochemical and immunologic methods. All isolates were stored at −80 °C in 10 % glycerol and grown in Luria–Bertani (LB) at 37 °C (Table 1).

**Table 1:** Source and virulence gene profiles of *E. coli* O157:H7 strains and other strains used in this study

No.	Strains	Presence of	Source	Identified
1	*E. coli* O157:H7	*Stx1*	ATCC	NIHE
2	*E. coli* O157:H7	*Stx2*	ATCC	HCMUS
3	*E. coli* O157:H7	*Stx1*, *Stx2*	ATCC	NLU
4	*Shigella sonnei*	–	ATCC	ATCC 9290
5	*Salmonella enterica*	–	ATCC	ATCC 14028
6	*Vibrio cholerae*	–	ATCC	ATCC 17802
7	*E. coli*	–	ATCC	ATCC 11775
8	*E. coli*	–	ATCC	ATCC 25922
9	*E. coli (n* = *8)*	–	Clinical isolate	E1–E8

*ATCC* American type culture collection

### Primer design

To design primers, central parts of shiga toxin genes were used as template. Features in the designed primers such as GC content, Tm, ΔG, etc. were checked by Oligo Explorer 1.1.0 and OligoAnalyzer 3.1 softwares. Two sets of primers are illustrated in Table 2. All primers were synthesized by Sigma company.

**Table 2:** Target genes and primer sequences used in this study

Target gene	GenBank accession *n*	Primer	Primer sequence (5′ → 3′)	PCR amplicon (bp)
*Stx1*	AB083043.1	*stx*1F	gag cga aat aat tta tat gtg	510
		*stx*1R	ttg atg atg gca att cag tat	
*Stx2*	JX161808.1	*stx*2F	cca tga caa cgg aca gca gtt	780
		*stx*2R	cct gtc aac tga gca ctt tgc	

### Sample collection

A survey was carried out with samples from local suppliers. Samples were transported to the laboratory, either frozen or refrigerated, and were kept under the same conditions until analysis. A total of 500 samples were analyzed which included seafood, vegetables, and meats, of about 100 g each, were collected from the refrigerators of the butcher department that served the hospital and samples of cooked meat were collected from the hospital’s kitchen just after cooking. Besides, some of them were also collected from different at markets of Ho Chi Minh City, Vietnam. All samples were transported to the laboratory at 4 °C and tested for *E coli* O157:H7 by both the traditional bacteriological method [ISO 16654 (2001)] and the multiplex PCR.

### Template DNA preparation after enrichment

One mL of overnight culture was transferred to a microcentrifuge tube and centrifuged 12,000×*g* for 3 min. The supernatant was removed and the pellet was resuspended in 1 mL of 0.85 % NaCl. After another centrifugation at 12,000×*g* for 3 min, the supernatant was removed and the pellet was resuspended in 1 mL of sterile water. After boiling at 100 °C for 10 min and being centrifuged at 12,000×*g* for 1 min, the supernatant was saved as a template DNA for PCR analysis.

### Direct culture method

*Escherichia coli* O157 detection and identification was determined by the reference method described by International Organization for Standardization ISO 16654 (2001). Briefly, 25 g of each sample was diluted in 225 mL of Modified Tryptone Soya broth (mTSB—Oxoid, UK) added with Novobiocin, homogenized for 2 min at 260 rpm using a Stomacher (Model 400 circulator, Seward, Norfolk, England) and incubated for 18–24 h at 41.5 °C according to ISO 16654 (2001) method, as well as the remaining steps. After enrichment and immunomagnetic concentration steps, the selective and differential isolation of enterohemorrhagic *E. coli* O157:H7 was carried out on MacConkey Agar with Sorbitol, Cefixime, and Tellurite (CT-SMAC—Oxoid, UK) and incubated overnight at 42 °C. From each sample one well isolated suspected colony was transferred to tryptone soy agar (Oxoid) and incubated for 24 h at 37 °C. Subsequently, one isolate from the subculture was further tested for agglutination with an *E. coli* O157:H7 latex test kit (Becton–Dickinson, USA) for serogroup O157:H7 confirmation.

### Isolation and amplification of *stx1* and *stx2* genes by PCR

PCR reagents in a final volume of 25 μL included: 1 μL template DNA, 0.5 μL Taq DNA polymerase (5 U/μL), 1 μL of each primer (10 pmol/μL), 1 μL dNTP mixture, 2.5 μL 10× PCR buffer, 1.5 μL MgCl_2_ (50 mM) and 16.5 μL sterile DDW. Thermal cycling of amplification mixture was performed in 30 cycles. The PCR program was carried out at 94 °C for 3 min followed denaturing for 45 s at 94 °C, annealing for 45 s at 54 °C and an extension for 1 min at 72 °C. The final extension was at 72 °C for 5 min. PCR products were electrophoresed in 1 % agarose followed by staining with ethidium bromide (0.5 g/mL) then visualized under ultraviolet light, and the results were recorded by photography. For labeling of PCR products, the reaction of PCR was performed by dNTP mixture containing digoxigenin labeling mix (Digoxigenin dNTPs, Roche, Germany) with the same condition.

### Validation of multiplex PCR methods for the detection of *E. coli* O157:H7 in food

ISO 16140 (2003) establishes the general principle and technical protocol for the validation of alternative methods in the microbiological analysis of food, animal feeding stuff, and environmental and veterinary samples. The validation protocol comprises two phases: the first is a method comparison study of the alternative method against the reference method [ISO 16654 (2001)], and the second is an interlaboratory study of each of the two methods. In the first step, ISO 16140 (2003) validation examines the limit of detection (LOD_50_); The second step entails the organization of an interlaboratory study, as described in Annex H of ISO 16140 (2003), to determine the following parameters: such as sensitivity, accuracy, specificity, the false positive rate and false-negative rate, its compared with culture method, respectively. The data reported here describe the results of validation, according to ISO 16140 (2003), of the reference method [ISO 16654 (2001)].

#### Determination of limit of detection (LOD_50_) following Spearman-Kaber method (Hitchins 2002; Kotz et al. 2006)

To calculate the LOD_50_ value, the process is performed as followed: (1) prepare 50 blank samples with the same matrix. To take 25 g for each sample (according to the validation method) into sterile plastic bag, then adds 225 mL modified Tryptone Soy Broth (mTSB); (2) a spiking inoculum was prepared starting from *E. coli* O157:H7 at a concentration of 10 CFU/mL: five binary series of dilutions (2^0^, 2^−1^, 2^−2^, 2^−3^, 2^−4^ CFU/mL) were prepared and five different food samples were spiked with 1 mL of each dilution; (3) after incubation, the pre-enriched samples were tested with the multiplex PCR detection as described above; LOD_50_ is determined as below:

$${ \log }\left( {{\text{LOD}}_{50} } \right) \, = \, L \, - \, d\left(\sum P_{i} {-} \, 0,5\right) \, = \, m;{\text{ or LOD}}_{50} = 10^{m}$$

with confidence limit 95 %, LOD_50_ is in range {10^*m*−2SQRT(*Um*)^; 10^*m*+2SQRT(*Um*)^} where LOD_50_ detection limit of the method, *L* log of lowest bacteria number that is spiked at this level where 100 % are positive results, *d* log of dilution concentration, *P*_*i*_ rate of positive results (50 % ≤ *Pi* ≤ 100 %) for the dilution concentration of *i*, *Um* estimation of measurement uncertainty for *m*, *n* number of repeatability test at the dilution concentration of *i* (*n* = 10).

#### Parameters evaluation of multiplex PCR method

According to ISO 16140 (2003), the parameters of the multiplex PCR method needed to evaluate such as relative accuracy (AC), relative sensitivity (SE) and relative specificity (SP), its compared with the reference method, respectively. A total of 60 test portions of third food categories were analyzed: 50 % of the samples were contaminated by *E. coli* O157:H7 at three levels (LOD, LOD × 10, LOD × 20); the other 50 % were negative samples. For the “vegetable-based products” category we evaluated 20 samples of lettuces, 18 of beans, 20 of spring onions, and 2 of watercress. For the “seafood product” category we analyzed 60 samples of seafood mix (previously tested negative for *E. coli* O157:H7) contaminated at the same concentrations as the “vegetable-based products” category. The relative detection level was determined after contamination of 60 meat products (chicken, pork and beef) with *E. coli* O157:H7 at three concentrations (20 negative controls, 20 samples spiked at LOD level, and 20 samples spiked at LOD × 3 level). Each combination (food product–level of contamination) was replicated six times with the alternative and reference methods for both food categories. Each combination (food product—level of contamination) was replicated six times with the alternative and reference methods for both food categories. For the “vegetable-based products” category we tested 60 samples while for “seafood products” category we analyzed 60 sample of seafood mix.

The inclusivity of the alternative method was evaluated by testing 50 pure cultures of target microorganism (*E. coli* O157:H7). The inoculums were 10–100 times greater than the minimum LOD_50_ of the alternative method. Exclusivity was verified by testing 30 pure cultures of non-target strains (50 % of *E. coli*, 50 % of other pathogenic strains consist of *Shigella sonnei* ATCC 9290, *Salmonella enterica* ATCC 14028 and *Vibrio cholerae* ATCC 17802). All samples of inclusivity and exclusivity were tested using only the alternative method. In adherence to the Annex H of ISO 16140 (2003) guidelines and requirements for the organization, dispatching, and conducting the interlaboratory study, we selected the third most relevant food matrices [according to Annex B of ISO 16140 (2003)] as the food matrix for the study: vegetable for “vegetables-based products”, seafood mix for “seafood products” and chicken, pork, beef for “meat products”. The samples were contaminated with *E. coli* O157:H7 and analyzed the blind samples with both the alternative and the reference method.

## Results

### Specificity of the primers to detect stx1 and stx2 genes by PCR

To assess the specificity of the primers *stx1F*/*stx1R* and *stx2F*/*stx2R* for the detection of genes coding for toxins *stx1* or *stx2*, the strains in Table 1 were used, which included 3 strains of *E. coli* O157:H7, 10 strains of non-*E. coli* O157:H7 and 3 other pathogenic bacteria (*Salmonella enterica* ATTC 14028, *Shigella sonnei* ATCC 9290 and *Vibrio cholerae* ATCC 17802).

**Fig. 1:**
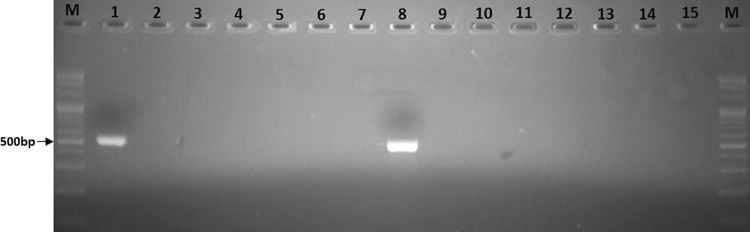
Specificity of primer pair *stx*1F/stx1R in the optimized PCR for amplification of *E. coli* O157:H7. *Lanes* M, 100-bp DNA ladder; 1, *E. coli* O157:H7 (NIHE); 2, *E. coli* ATCC 25922; 3, *E. coli* 11775; 4, *Salmonella enterica* 14028; 5, *Shigella sonnei* 9290; 6, *Vibrio cholera* 17802; 7, Water; 8, *E. coli* O157:H7 (NLU); 9–15, the strains of E1–E7, respectively

In the primers *stx1*, the presence of amplified products size 520 bp after agarose gel electrophoresis. Reliable amplification of band of *stx1* was obtained in standard *E. coli* 0157:H7 strains. As a negative control PCR was tested with other strains and water no amplicons were observed (Fig. 1).

**Fig. 2:**
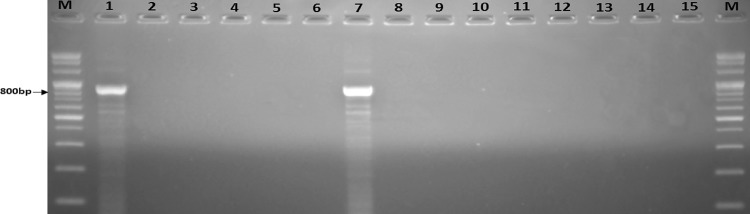
Specificity of primer pair stx2F/stx2R in the optimized PCR for amplification of *E. coli* O157:H7. *Lanes* M, 100-bp DNA ladder; 1, *E. coli* O157:H7 (HCMUS); 2, *E. coli* ATCC 25922; 3, *E. coli* ATCC 11775; 4, *Salmonella enterica* ATCC 14028; 5, *Shigella sonnei* ATCC 9290; 6, *Vibrio cholerae* ATCC 17802; 7, *E. coli* O157:H7 (NLU); 8, Water; 9–15, the strains of E1–E7, respectively

On the other hand, in the primers *stx2*, the presence of amplified products size 780 bp after agarose gel electrophoresis. Reliable amplification of band of *stx2* was obtained in standard *E. coli* 0157:H7 strains. As a negative control PCR was tested with other strains and water no amplicons were observed (Fig. 2).

### Establishment of the mutiplex PCR methods to detect *E. coli* O157:H7 based on *stx*1 and *stx*2 gene

**Fig. 3:**
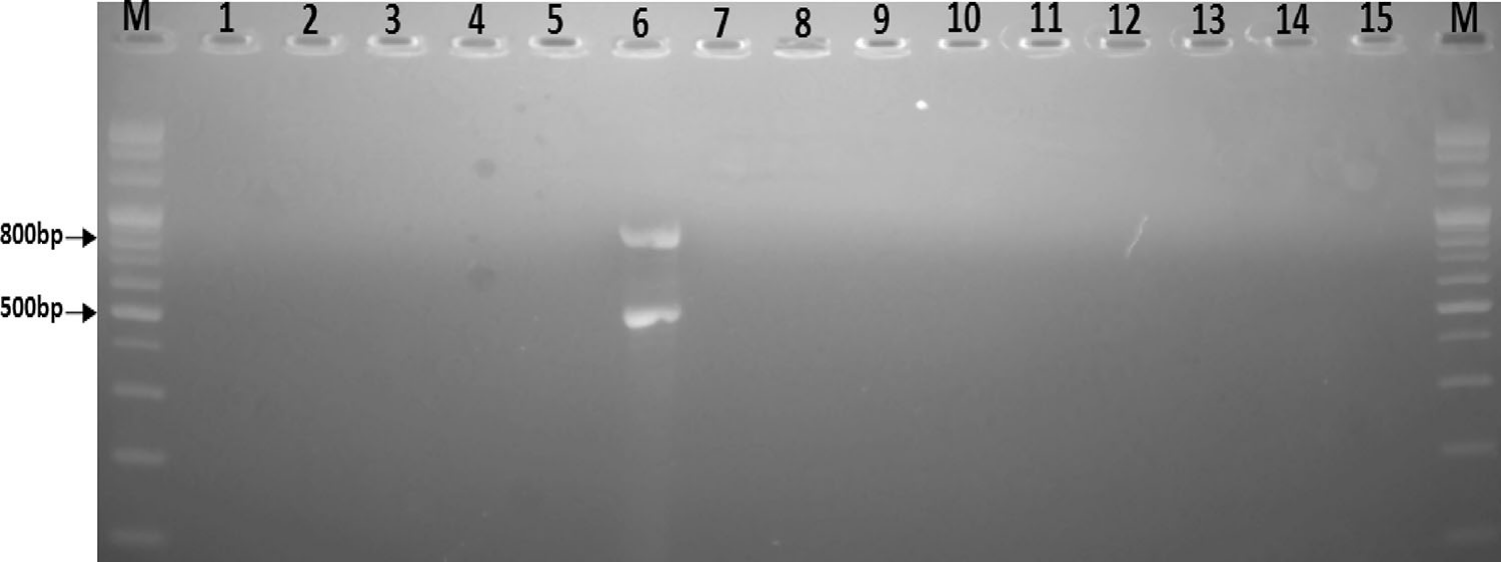
Specificity of primer pair stx1, stx2 in the optimized PCR for amplification of *E. coli* O157:H7. *Lanes* MR, 100-bp DNA ladder; 1, *E. coli* ATCC 25922; 2, *E. coli* ATCC 11775; 3, *Salmonella enterica* ATCC 14028; 4, *Shigella sonnei* ATCC 9290; 5, *Vibrio cholerae* ATCC 17802; 6, *E. coli* O157:H7 (NLU); 7, Water; 8–15, the strains of E1 to E8, respectively

Shiga toxins are produced by *E. coli* O157:H7 strains. Shiga toxin might be produced from strains carrying *stx1* or *stx2* gene only or both. Therefore, we have combined two primers for simultaneous detection of *stx*1 and *stx*2 in the same PCR amplification reaction. The reaction conditions for the multiplex PCR assay were optimized to ensure that all of the target gene sequences were satisfactorily amplified. The primers were designed with care to avoid areas of homology with other organisms. The primers had almost equal annealing temperature, which reduced the possibility of nonspecific amplification. The annealing temperature of 54 °C was finally selected based on nearly equal intensity of PCR products. Figure 3 shows the presence of amplified products size 520 and 780 bp after agarose gel electrophoresis. Reliable amplification of two bands of *stx1* and *stx2* was obtained in standard *E. coli* 0157:H7 strain (NLU). As a negative control multiplex PCR as water, other microbiologisms were tested no amplicons were observed.

#### The results of limit of detection (LOD_50_)

The LOD_50_ was performed with 50 samples, including vegetable, seafood mix and meat products. Blank samples used for validation are without *E. coli* O157:H7 contamination. These samples are considered negative control samples and used for spiking target bacteria for validation purposes. In this case, the strain of *E. coli* O157: H7 carrying *stx1* and *stx2* from Nong Lam University (NLU) was selected for contaminated to sample. The analysis as the multiplex PCR has been established, results show in Table 3. Appling to the formula above, the LOD of the multiplex PCR method was set at 3 CFU/25 g or mL for third food categories.

**Table 3:** The results of LOD_50_ are summarized

Concentration of *E. coli* O157 in 25 g (CFU)	Dilution factor	No. of test	No. of test with positive results	Rate of positive
10		10	10	1
6		10	9	0.9
4	2	10	7	0.7
3		10	5	0.5
2		10	3	0.3

*CFU* colony-forming unit

#### The results of validation of the technical parameters of newly mPCR methods

These parameters were determined for third groups of samples consist of vegetables-based products, seafood products and meat products. Carry out 60 samples for each same group samples 30 samples are contaminated by *E. coli* O157:H7 and 30 samples are contaminated by other pathogenic bacteria. The relative accuracy, specificity and sensitivity of each food matrix are reported in Table 4.

**Table 4:** Calculation of the technical parameters of newly multiplex PCR method for detection of *E. coli* O157:H7

Matrices	PA	NA	ND	PD	Sum	Relative accuracy AC (%)	*N*+	Relative sensitivity SE (%)	*N*−	Relative specificity SP (%)
					*N*	*100* × *(PA* + *NA)/N*	*PA* + *ND*	*100* × *PA/N*+	*NA* + *PD*	*100* × *NA/N*−
Vegetables-based products	28	30	0	2	60	100 × (28 + 30)/60	28	100 × 28/28	32	100 × 30/32
						96.66 %		100 %		93.75 %
Seafood products	28	30	0	2	60	100 × (28 + 30)/60	28	100 × 28/28	32	100 × 30/32
						96.66 %		100 %		93.75 %
Meat products	28	30	0	2	60	100 × (28 + 30)/60	28	100 × 28/28	32	100 × 30/32
						96.66 %		100 %		93.75 %

Relative accuracy, sensitivity and specificity were calculated as follows: accuracy AC = ((PA + NA)/*N*) × 100 %; specificity SP = (NA/*N*) × 100 %; sensitivity: SE = (PA/*N*) × 100 %, where PA is the agreement for positive results; NA is the agreement for negative results; *N* is the total number of samples; *N*_−_ is the total number of negative results with the reference method (*N*_−_ = NA + PD); *N*_+_ is the total number of positive results with the reference method (*N*_+_ = PA + ND); PD is positive deviation (i.e., false positive result); ND is negative deviation (i.e., false negative result)

These values were set considering a 95 % confidence interval (CI) with different lower confidence limits (LCL): calculation of the confidence intervals is associated with the number of samples tested, as specified in Annex E of ISO 16140 (2003). In particular, only two product samples of each food category have resulted positive with the alternative method and negative with the reference method. For each level and each food/strain combination, the two methods were compared using Fisher’s exact test: a *p* value = 1 was obtained for third food categories.

## Discussion

*E. coli* O157:H7 is a worldwide threat to public health and the outbreaks historically have been associated with undercooked ground beef, meat and meat products, raw vegetables. *E. coli* O157:H7 strains possessing important virulence traits are required to be surveyed particularly when the strain is involved in disease outbreaks to assess the response strategies for containment. *E. coli* O157:H7 detection and identification was determined by the reference method described by International Organization for Standardization ISO 16654 (2001), which involves several steps of enrichment, plate isolation and biochemical identification, taking up to 4–6 days in the case of *E. coli* O157:H7. However, PCR-based techniques have the potential to allow for rapid and sensitive detection of foodborne pathogens. Since PCR can target unique genetic sequences such as virulence genes of microorganisms, it also has the advantage of potentially being an extremely specific assay (Fratamico and Strobaugh 1998). In addition, many authors have demonstrated the advantages of using multiplex PCR methods (Elizaquivel and Aznar 2008; Ruiz-Rueda et al. 2011; Singh et al. 2011). In this study, we developed a rapid, reliable and specific mPCR method to successfully detect *E. coli* O157:H7 in food by analyzing the two major virulence genes. The primers used in the present study were designed to specifically amplify *Stx1*, *Stx2* gene of *E. coli* O157:H7, respectively. Additional, specificity of the primers to detect *stx1* and *stx2* genes as well examined on strains non-*E. coli* O157:H7, to achieving as expected. Meanwhile, the process of multiplex PCR involves designing each primer set in a single PCR mixture to amplify amplicons that are specific to the target DNA sequences. Furthermore, the annealing temperature of the multiplex PCR reaction is optimized to achieve distinct bands for each primer sets. For the mPCR reported here, all the primers were designed with the view to have a common annealing temperature to get preferable amplification at a single temperature and care was also taken to maintain at least 100 bps differences between product sizes for good resolution during agarose gel electrophoresis. Our study portrayed the best amplicons at high annealing temperature of 54 °C (Fig. 3).

Bacterial pathogens may coexist, at different concentrations, in the same food sample, but they usually occur at low levels. Their detection is usually preceded by an enrichment step to increase cell numbers to the detection level. Hence, enrichment is necessary to obtain a higher number of positive results by mPCR with *E. coli* O157:H7. The adequacy of mPCR in identifying *E. coli* 0157:H7 strains in general and the toxin containing strains in particular following overnight enrichment of food and environmental samples in mTSB broth was well established. The *stx1* and *stx2* genes being responsible for the expression of the potential toxin molecules can be easily be identified by the reported mPCR and this protocol would help in detecting them during biological emergencies. Considering the low cost and the associated rapidity to detect the four genes simultaneously, it is believed that this may serve as a powerful tool for not only to obtain a reliable identification of *E. coli* 0157:H7, but also in assessing the toxin potential of the strain as well. The procedure is especially suited to fit into the daily work requirement of routine food quality control laboratories since detection and identification of the toxin genes of the pathogen from diverse food sources is becoming an important component of the diagnostic inventory of such laboratories. Given the long turnaround time associated with traditional culture methods, PCR in any case is a rapid and reliable screen for the detection of *E. coli* 0157:H7 harboring *stx1* and *stx2* genes. These species are long-term and highly sensitive at different culture conditions: using DNA as the target for sample screening overcomes problems with culture conditions, allowing the rapid identification of positive samples, with the identification of a possible *E. coli* O157:H7 source within 48 h. By contrast, the reference method [ISO 16654 (2001)] requires at least 4 days for determining negative samples and more than 7 days for species identification. Also, the laboratory in Vietnam analysis of *E. coli* O157:H7 in foods mainly based on the conventional culture method. Therefore, the analysis time is 4–7 days, and not conformity with the requirements for fast analysis of food samples suspected *E. coli* O157:H7 serve the purpose of monitoring emergency.

The results of this study show that mPCR is useful to detect and identify *E. coli* O157:H7 in food, even if the concentration of bacteria is low (2–3 cells of bacteria/25 g of food). Incubation of the homogenized food in mTSB at 41.5 or 37 °C for at least 10 h is necessary to archive the levels of enrichment for reliable detection of this pathogen in food. The study also shows that the mPCR system has comparable sensitivity to the direct culture method and would be amenable for on-site analysis of food by marginally skilled personnel, since it does not require the same level of training to perform as does the more rigorous multiplexed PCR technique described described here although there is evident that both used methods characterize distinct specificity and that they are not completely replaceable.

In conclusion, the multiplex PCR method with two pairs of primers for amplifying the *stx1*, *stx2* target genes to detect *E. coli* O157:H7 in food has been set up successfully. The novel method is developed with the following basic parameters: enrichment of 25 g samples into sterile bags already containing 225 mL mTSB, incubate at 41.5 °C for 10–24 h. One milliliter enrichment solution was taken at the end of incubation period and processed for DNA extraction by boiling method. The DNA was used as template in multiplex PCR assay with primers *stx1F*/*stx*1R and *stx*2F/*stx*2R, at concentration 0.5 μM of each primer. The final concentration of MgCl_2_ is 2.5 μM. The results of evaluating of method show that the multiplex PCR method has LOD is 3 CFU/25 g or mL sample, sensitivity is 100 %, accuracy is 96.66 %, specificity is 93.75 %. A newly developed multiplex PCR assay for the detection of *E. coli* O157:H7 based on shiga-like toxin genes *stx1* and *stx2*, have indicated all the criteria and parameters tested were met according to laboratory requirements. Our data suggest that this commercial kit is suitable for use in official controls on vegetable, seafood and dairy product food samples. Moreover, because multiplex PCR-based methods are fast and easier for laboratory testing, they can identify the likely microbiological source in 48 h. The rapid response time could help public health authorities in diagnosing and treating foodborne illnesses early caused by *E. coli* O157:H7. Finally, this approach could provide timely data for updating report database systems with information about risk factors.
